# Antimicrobial susceptibility profiles of *Escherichia coli* isolates from domestic pigeons in Hungary

**DOI:** 10.3389/fvets.2025.1642902

**Published:** 2025-09-12

**Authors:** Ádám Kerek, Ábel Szabó, Ákos Jerzsele

**Affiliations:** ^1^Department of Pharmacology and Toxicology, University of Veterinary Medicine Budapest, Budapest, Hungary; ^2^National Laboratory of Infectious Animal Diseases, Antimicrobial Resistance, Veterinary Public Health and Food Chain Safety, University of Veterinary Medicine Budapest, Budapest, Hungary

**Keywords:** antimicrobial resistance, *Escherichia coli*, minimum inhibitory concentration, multidrug resistance, pigeons

## Abstract

**Background:**

This cross-sectional observational study aimed to determine the antimicrobial susceptibility profiles of *Escherichia coli* isolates from domestic pigeons (Columba livia domestica) in Hungary.

**Methods:**

A total of 134 non-redundant isolates were obtained from oropharyngeal and cloacal swabs collected across multiple geographic regions. Antimicrobial susceptibility testing was conducted using the microdilution method, following Clinical and Laboratory Standards Institute (CLSI) guidelines. Minimum inhibitory concentration (MIC) values were determined for a panel of antibiotics relevant to both veterinary and human medicine. Resistance patterns were analyzed using statistical tools including hierarchical clustering, network graph analysis, decision tree modeling, and Monte Carlo simulation.

**Results:**

Multidrug-resistant (MDR) strains constituted 65.7% of the total, while extensive drug-resistant (XDR) and pan-drug-resistant (PDR) strains were found in 4.5% and 1.5% of isolates, respectively. The highest resistance rates were observed for neomycin (76.1%) and florfenicol (72.4%), whereas ceftriaxone and imipenem showed the lowest resistance rates (0.7% and 1.5%). Correlation analysis indicated notable associations between resistance to neomycin, doxycycline, and florfenicol, suggesting potential cross-resistance mechanisms. Monte Carlo simulation estimated an average MDR prevalence of 64.4% (95% CI: 50.0–77.6%). The high prevalence of antimicrobial resistance among pigeon-derived *E. coli* isolates underscores the potential public health risks posed by avian reservoirs within the One Health context.

**Conclusions:**

These findings highlight the need for enhanced AMR surveillance and responsible antibiotic stewardship in veterinary settings. Further molecular investigations are warranted to elucidate the genetic basis of resistance in this population.

## 1 Introduction

The discovery of antibiotics brought about a revolutionary change in medicine. Since the identification of the first patented antimicrobial agent by Salvarsan in 1910 (arsphenamine), antibiotics have been used to treat various bacterial infections (1). However, due to the rapid adaptability of bacteria, resistance mechanisms have begun to emerge (2, 3). Initially, many believed that the primary driver of antibiotic resistance was the overuse of antibiotics in human medicine (4). However, it has become increasingly evident that the agricultural sector, particularly veterinary medicine, also act as significant risk factors and determinants in the emergence and dissemination of antimicrobial resistance (5). In this context, antibiotic usage should be considered a predisposing factor rather than a direct source or reservoir of resistance. This issue was especially problematic with the long-term subtherapeutic application of antibiotics, particularly when used as growth promoters—a practice that has been banned in the European Union, including Hungary, since 2006. However, recent studies indicate that such use may still persist in certain regions worldwide (6). Due to the widespread dissemination of resistance, greater emphasis must be placed on the prudent use of antibiotics (7) and the exploration of partial or complete replacement with various alternative strategies (8–15).

The role of pigeons (*Columba livia domestica*) in urban environments deserves special attention when studying the spread of antimicrobial resistance (16). These birds are domesticated descendants of the rock pigeon (*Columba livia*) (17), and approximately 124 breeds and varieties have been selectively bred and documented worldwide (18). In Hungary, pigeons are widely kept for various purposes including racing, ornamental breeding, and hobby purposes, with some being sold at local markets. While no formal national registry exists, unofficial estimates suggest that tens of thousands of pigeons are kept in urban and peri-urban areas, often in close proximity to both humans and other domesticated or wild bird species such as sparrows, starlings, or doves, which are also potential AMR carriers. Pigeons frequently come into contact with animal feces, contaminated water sources, and refuse, creating multiple opportunities for the acquisition and dissemination of resistant bacteria (19). Moreover, the potential zoonotic transmission of antimicrobial-resistant strains from pigeons to humans poses a significant public health risk, especially in urban areas where close contact with humans is common. During interactions with humans, they may act as environmental reservoirs and mechanical vectors for AMR microorganisms, primarily through fecal shedding and environmental contamination, thereby contributing to the urban AMR burden (20, 21).

Despite their ubiquity and close contact with human settlements, pigeons remain an understudied source of antimicrobial-resistant bacteria, particularly in Central and Eastern Europe. Most existing AMR surveillance frameworks focus on poultry, swine, and cattle, while synanthropic avian species are rarely included, leaving critical gaps in our understanding of environmental and urban AMR dynamics. To date, limited data are available on the antimicrobial susceptibility of *E. coli* isolates from pigeons in Hungary, and the potential public health implications of such strains remain largely unexplored. This study aims to fill that gap by providing a detailed profile of *E. coli* resistance patterns in domestic pigeons, which may serve as both environmental reservoirs and passive vectors for resistant bacteria.

Environmental conditions may further contribute to the selection and persistence of resistant strains. The presence of antibiotics in environmental sources, including drinking water and rainwater storage tanks, exerts selective pressure on microbial communities, supporting the survival of resistant organisms (19). Bird droppings on rooftops, in particular, can contaminate these water sources and elevate the risk of zoonotic gastrointestinal infections (22). The detection of *E. coli* in such settings is commonly used as an indicator of fecal contamination and potential AMR risk (23).

One of these bacteria is *Escherichia coli* (*E. coli*), a rod-shaped, Gram-negative, facultative anaerobic bacterium commonly found in the gastrointestinal tracts of warm-blooded animals, particularly mammals and birds, as well as in various environmental reservoirs (24). In birds, several pathogenic *E. coli* pathotypes have been identified, including avian pathogenic *E. coli* (APEC), enteropathogenic *E. coli* (EPEC), and Shiga toxin-producing *E. coli* (STEC). APEC is associated with extraintestinal infections such as septicemia, pericarditis, and salpingitis, and has demonstrated genetic and virulence similarities to human ExPEC strains, suggesting zoonotic potential (25–29). EPEC and STEC, both known for their zoonotic relevance, can be transmitted to humans through contaminated meat or direct contact, posing significant public health concerns (22, 23, 30).

The use of antibiotics in veterinary medicine is essential for treating *E. coli* infections. However, zoonotic *E. coli* strains originating from birds are ubiquitous in the environment and can be transmitted to humans not only through contaminated meat, but also via fruits, vegetables, water, and surfaces, posing a broad-spectrum public health risk. Doxycycline, a broad-spectrum tetracycline, acts by inhibiting bacterial protein synthesis (31–33). In domesticated and racing pigeons, particularly in Hungary, antibiotics are frequently used for the treatment of chlamydiosis, conjunctivitis, and mycoplasmosis (34–36). Such treatments are not typically applied to wild pigeon populations. Florfenicol, a bacteriostatic antibiotic that acts by binding to the 50S ribosomal subunit, is effective against numerous Gram-negative pathogens, including *E. coli* (37, 38). Its efficacy through oral administration has also been demonstrated in poultry (39–41). Enrofloxacin, a fluoroquinolone antibiotic, inhibits bacterial DNA gyrase and is commonly used to treat gastrointestinal and urinary tract infections in birds (42, 43). In pigeons, it is used to treat salmonellosis and mycoplasmosis (44, 45). Colistin, also known as polymyxin E, belongs to the group of polypeptide antibiotics and has been used for a long time to treat infections caused by Gram-negative bacteria (46–48). Its mechanism of action involves altering the permeability of the bacterial cell membrane (49). Colistin plays a particularly important role in therapies targeting multidrug-resistant strains and is used in pigeons to treat enteritis caused by *Salmonella*, *Pasteurella*, and *E. coli* (50).

Understanding the susceptibility profiles of *E. coli* isolates to these commonly applied antibiotics is essential for mapping the epidemiological landscape of antimicrobial resistance, supporting risk assessment, and informing evidence-based therapeutic decisions in veterinary practice. While such data provide indirect insight into potential zoonotic risks, confirmation of transmission pathways requires genomic or molecular evidence.

The presence of antibiotics in environmental sources, including drinking water, exerts selective pressure on microbial communities, thereby facilitating the emergence and persistence of AMR. Although environmental reservoirs do not directly disseminate AMR, they can support the survival and proliferation of resistant strains that may later be transmitted through biological vectors or contact networks (19). In some regions, rainwater is commonly used for drinking purposes; however, it is often collected in tanks where contamination may occur. Bird droppings on rooftops can further elevate the risk of AMR transmission, potentially leading to zoonotic gastrointestinal infections (22). The detection of *E. coli* is frequently used as an indicator of fecal contamination in such water sources (23).

The aim of this study was to determine the antimicrobial susceptibility profiles of *E. coli* isolates from pigeons, collected in Hungary between 2022 and 2023. To contextualize these findings, resistance data from human *E. coli* isolates were also examined and compared descriptively. Particular attention was paid to the occurrence and co-resistance patterns of antibiotic resistance and the investigation of resistance mechanisms to individual antibiotics. The findings contribute to a better understanding of the role of pigeons in the spread of antimicrobial resistance and provide guidance for developing future veterinary and public health strategies under the One Health framework.

In addition to characterizing resistance in pigeon-derived *E. coli* isolates, the study also incorporates comparative data from human clinical isolates provided by the Hungarian National Public Health Service. This comparative perspective aims to contextualize resistance patterns observed in pigeons within the broader One Health framework. However, it is important to note that the human data were not collected prospectively but were included for descriptive and interpretative purposes.

## 2 Materials and methods

### 2.1 The origin of the strains

This study was designed as a cross-sectional observational investigation conducted between 2022 and 2023. The study population included domesticated pigeons (*Columba livia domestica*), kept for racing, hobby, or breeding purposes across multiple Hungarian regions. Samples were collected by a licensed veterinary practitioner during routine diagnostic visits, ensuring that sample collection reflected real-world clinical settings.

Swab samples were collected from 20 independent pigeon flocks, located in various Hungarian regions: Észak-Magyarország (3 flocks), Észak-Alföld (2), Dél-Alföld (8), Közép-Magyarország (5), Közép-Dunántúl (1), Dél-Dunántúl (1), and Nyugat-Dunántúl (2). Flocks were selected based on availability, prior veterinary contact, and geographical distribution to ensure diverse regional representation. Inclusion criteria included clinically healthy birds over 6 months of age, with no antimicrobial treatment administered in the 2 weeks prior to sampling. Birds showing clinical signs of disease or with unknown treatment status were excluded. Oropharyngeal and cloacal swabs were collected using Amies-type transport medium without charcoal (Biolab Zrt., Budapest, Hungary), employing standard aluminum shaft swabs. Bacterial identification was performed on ChromoBio Coliform agar. Pure cultures were stored in Microbank systems at −80 °C for further testing.

A total of 134 non-redundant *E. coli* isolates were included in the analysis. The sample size was pragmatically determined based on field access and logistical feasibility and was considered sufficient for robust statistical evaluation of antimicrobial resistance patterns. Isolates were selected based on colony morphology, sample type, and flock of origin to reduce overrepresentation and maximize epidemiological diversity within the constraints of feasible strain isolation and characterization.

### 2.2 Minimum inhibitory concentration (MIC) determination

The antibiotics selected for testing were chosen based on their documented and routine use in veterinary medicine, particularly in the treatment of bacterial infections in domesticated pigeons in Hungary. All pigeons sampled in this study were domesticated birds, kept for breeding, sport (racing), or ornamental purposes. They were managed under controlled conditions and housed in lofts or pens. None of the sampled birds were wild or free ranging. The panel included agents relevant to both avian clinical use and public health concern. Phenotypic resistance expression was assessed by determining MIC values following the Clinical Laboratory Standard Institute (CLSI) guidelines (51). Breakpoints were also established according to CLSI recommendations (51) and were compared with EUCAST-defined ECOFF values (52). For certain antibiotics not included in CLSI or EUCAST breakpoint tables—such as amoxicillin-clavulanate (53), neomycin (54), spectinomycin (53), and colistin (55), additional interpretive criteria were sourced from relevant peer-reviewed literature.

Prior to testing, bacterial strains stored at −80 °C were suspended in 3 mL of cation-adjusted Mueller-Hinton broth (CAMHB) and incubated for 18–24 h at 37 °C. Testing was performed using 96-well microtiter plates (VWR International, LLC., Debrecen, Hungary). All wells, except those in the first column, were filled with 90 μL of CAMHB. Stock solutions of tested agents (Merck KGaA, Darmstadt, Germany) were prepared at a concentration of 1,024 μg/mL according to CLSI guidelines (56).

Amoxicillin and amoxicillin-clavulanate were prepared in a 2:1 ratio (pH 7.2, 0.01 mol/L) and imipenem in phosphate buffer (pH 6, 0.1 mol/L). Doxycycline, neomycin, tylosin, and vancomycin were dissolved in distilled water. For the preparation of trimethoprim and sulfamethoxazole (1:19 ratio), sulfamethoxazole was dissolved in hot water with a few drops of 2.5 mol/L NaOH, while trimethoprim was dissolved in distilled water containing 0.05 mol/L HCl. Enrofloxacin was prepared by dissolving the compound in distilled water with the addition of 100 μL of 1 mol/L NaOH. Florfenicol was dissolved in distilled water with the addition of 100 μL of 95% ethanol to enhance solubility.

From these solutions, a 512 μg/mL dilution was prepared and 180 μL was added to the first column of the microtiter plates. A two-fold serial dilution was carried out across the plate, discarding 90 μL of the final well to ensure each well contained 90 μL. Bacterial suspensions adjusted to 0.5 McFarland standard (using a Nephelometer, ThermoFisher Scientific, Budapest, Hungary) were inoculated at 10 μL per well starting from column 11 and moving backward (51).

Assessment of the MICs was performed using a Sensititre™ SWIN™ automatic MIC reader and VIZION system software vs. 3.4 (ThermoFisher Scientific, Budapest, Hungary, 2024). The quality control reference strain used was *Escherichia coli* (ATCC 25922). This method ensures precise and reproducible determination of MIC values, allowing for consistent comparison with international standards.

MIC₅₀ and MIC₉₀ values were calculated as the 50th and 90th percentiles of the MIC distribution across all tested isolates, respectively. These values represent the MIC at which 50 and 90% of the isolates are inhibited. MIC distributions were obtained from the full dataset of 134 *E. coli* isolates tested against each antibiotic, and percentile thresholds were computed using base R functions. These metrics provide an aggregate view of susceptibility trends within the bacterial population.

Human *E. coli* resistance data were obtained from aggregated hospital surveillance records, provided with the formal permission of the Chief Medical Officer of Hungary. The data were supplied in the form of an Excel spreadsheet, containing regional and national resistance percentages for selected antibiotics. These records did not include isolates from pigeon handlers or consumers but rather represented general clinical cases reported through Hungary’s national surveillance system.

### 2.3 Statistical analysis

All statistical analyses were conducted using the R programming language (version 4.2.2) in the RStudio environment (57). The purpose of these analyses was to identify statistically significant resistance patterns, groupings among isolates, and cross-resistance trends, while also enabling simulation-based extrapolation of MDR prevalence. The Shapiro–Wilk test was used to assess data normality. For data not following normal distribution, non-parametric tests were chosen to ensure robust inference. The Kruskal-Wallis test (58) was used to compare the extent of resistance among various antibiotic classes. This test does not assume normal distribution and is ideal for comparing median differences across multiple groups. *Post hoc* comparisons were performed using the Mann–Whitney U test (59), while Welch’s t-tests were used for normally distributed subsets. Bonferroni correction was applied to adjust for multiple comparisons and reduce Type I error, acknowledging a higher chance of Type II error (60).

Because resistance data were binarized (resistant = 1, susceptible = 0), we used the phi coefficient instead of Pearson’s r to assess pairwise associations between antibiotics. The phi coefficient is specifically suited for binary categorical variables and was computed using the psych package in R. Correlations were interpreted using thresholds of |*φ*| ≥ 0.7 as strong, 0.4–0.69 as moderate, and 0.2–0.39 as weak. Correlation analysis among antibiotics was visualized using heatmaps generated by the corrplot (v0.92) and pheatmap (v1.0.12) packages.

Agglomerative hierarchical clustering was performed using the cluster (v2.1.4) package with Jaccard distance and Ward’s linkage method. For visualization, principal component analysis (PCA) was applied to the binarized matrix using the factoextra (v1.0.7) package, and the resulting clusters were projected into PCA coordinate space. Dendrograms were visualized using the dendextend (v1.16.0) package.

Network analysis was used to identify frequent co-resistance connections. Networks were generated with igraph (v1.3.5) and visualized using ggraph (v2.1.0). Edges represented joint resistance between antibiotic pairs, weighted by co-occurrence frequency, and were interpreted structurally, not inferentially.

For Pearson correlation, resistance data were binarized based on clinical breakpoints, and pairwise correlation coefficients were calculated between antibiotics to evaluate similarity in resistance profiles across isolates. For the Network Graph Analysis, connections were established between antibiotics based on the number of isolates exhibiting resistance to both agents, with edge weights reflecting co-occurrence frequency. Unlike Pearson correlation, this method did not assess statistical dependence but rather the structural presence of joint resistance traits.

To model potential predictors of MDR phenotypes, we used decision tree models (rpart, v4.1.16) and validated their performance with caret (v6.0.93).

To assess the uncertainty around the observed MDR prevalence, we performed bootstrap resampling (10,000 iterations) using the boot package (v1.3.28) in R. This approach generated an empirical distribution of MDR proportions, from which a 95% confidence interval was derived. Results were visualized using the ggplot2 package (v3.4.0).

### 2.4 Definitions of resistance categories

The classification of multidrug-resistant (MDR), extensively drug-resistant (XDR), and pan-drug-resistant (PDR) *E. coli* strains followed the standardized definitions proposed by Magiorakos et al. (61). MDR was defined as acquired non-susceptibility to at least one agent in three or more antimicrobial categories. XDR was defined as non-susceptibility to at least one agent in all but two or fewer categories (i.e., bacterial isolates remain susceptible to only one or two categories). PDR was defined as non-susceptibility to all agents in all antimicrobial categories tested. These criteria were applied after categorizing isolates based on MIC values and corresponding CLSI-defined clinical breakpoints (61).

## 3 Results

### 3.1 Study population characteristics

A total of 660 swab samples (330 cloacal and 330 oropharyngeal) were collected from 330 domestic pigeons. *E. coli* was detected in 328 samples (49.7%). Most of the isolates originated from the Dél-Alföld region (52.2%). The majority of isolates (32.8%) were obtained from racing pigeons, with most of the pigeons being young adults (32.8%) and older, breeding birds (28.4%). The majority of pigeon keepers (47.8%) maintained medium-sized flocks (51–100 birds).

**Figure 1:**
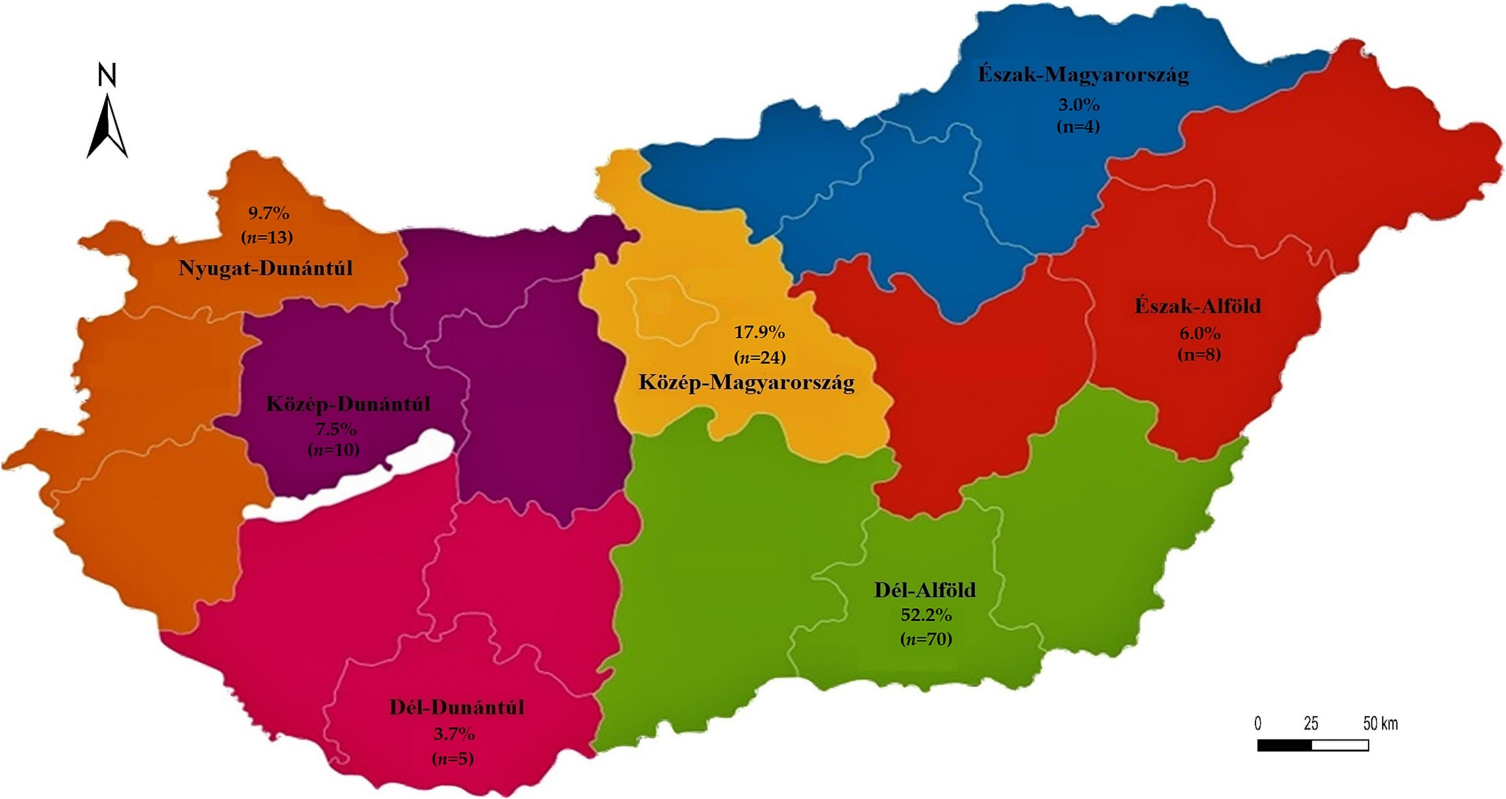
Regional distribution of *Escherichia coli* isolates (*n* = 134) from pigeons. The map displays the proportion (%) and absolute number (*n*) of isolates per administrative region. The map was generated manually using Adobe Illustrator. Regional boundaries correspond to the official Hungarian NUTS-2 level divisions. The different colors serve to visually distinguish between regions.

Figure 1 presents the geographical distribution of the isolates. A total of 134 *E. coli* isolates were obtained from cloacal and oropharyngeal swabs of pigeons sampled from 20 different flocks across Hungary. The study population included birds from seven regions, with the highest sampling density in Dél-Alföld and Közép-Magyarország. Most pigeons were reared for hobby or sport (e.g., racing) purposes, while others were kept for breeding. Flock sizes ranged from 15 to 150 birds, and the age distribution of sampled individuals spanned 6 months to over 4 years. A detailed breakdown of sample numbers by region, purpose of rearing, and age category is provided in Tables 1–3.

**Table 1:** Distribution of sampled pigeon flocks by region and primary purpose of keeping.

Region	Homing, pcs (%)	Meat, pcs (%)	Ornamental, pcs (%)
Dél-Alföld	26 (19.4%)	21 (15.7%)	23 (17.2%)
Közép-Magyarország	11 (8.2%)	0 (0.0%)	13 (9.7%)
Észak-Alföld	3 (2.2%)	0 (0.0%)	5 (3.7)
Dél-Dunántúl	0 (0.0%)	5 (3.7)	0 (0.0%)
Észak-Magyarország	4 (3.0%)	0 (0.0%)	0 (0.0%)
Közép-Dunántúl	10 (7.5%)	0 (0.0%)	0 (0.0%)
Nyugat-Dunántúl	13 (9.7%)	0 (0.0%)	0 (0.0%)

**Table 2:** Distribution of sampled pigeon flocks by region and predominant age category of animals.

Region	Shipped, pcs (%)	Young, pcs (%)	Adult, pcs (%)	Breeding, pcs (%)
Dél-Alföld	17 (12.7%)	21 (15.7%)	21 (15.7%)	11 (8.2%)
Közép-Magyarország	0 (0.0%)	1 (0.7%)	13 (9.7%)	10 (7.5%)
Észak-Alföld	0 (0.0%)	6 (4.6%)	1 (0.7%)	1 (0.7%)
Dél-Dunántúl	0 (0.0%)	2 (1.5%)	1 (0.7%)	2 (1.5%)
Észak-Magyarország	0 (0.0%)	1 (0.7%)	3 (2.2%)	0 (0.0%)
Közép-Dunántúl	0 (0.0%)	0 (0.0%)	0 (0.0%)	10 (7.5%)
Nyugat-Dunántúl	0 (0.0%)	2 (1.5%)	7 (5.2%)	4 (3.0%)

**Table 3:** Distribution of sampled pigeon flocks by region and flock size category.

Region	1–50 pcs (%)	51–100 pcs (%)	101–500 pcs (%)	>501 pcs (%)
Dél-Alföld	22 (16.5%)	25 (18.7%)	2 (1.5%)	21 (15.7%)
Közép-Magyarország	0 (0.0%)	21 (15.7%)	3 (2.2%)	0 (0.0%)
Észak-Alföld	0 (0.0%)	0 (0.0%)	8 (5.9%)	0 (0.0%)
Dél-Dunántúl	0 (0.0%)	0 (0.0%)	5 (3.7)	0 (0.0%)
Észak-Magyarország	0 (0.0%)	1 (0.7%)	3 (2.2%)	0 (0.0%)
Közép-Dunántúl	0 (0.0%)	10 (7.5%)	0 (0.0%)	0 (0.0%)
Nyugat-Dunántúl	0 (0.0%)	13 (9.7%)	0 (0.0%)	0 (0.0%)

### 3.2 Resistance correlations and clustering

**Figure 2:**
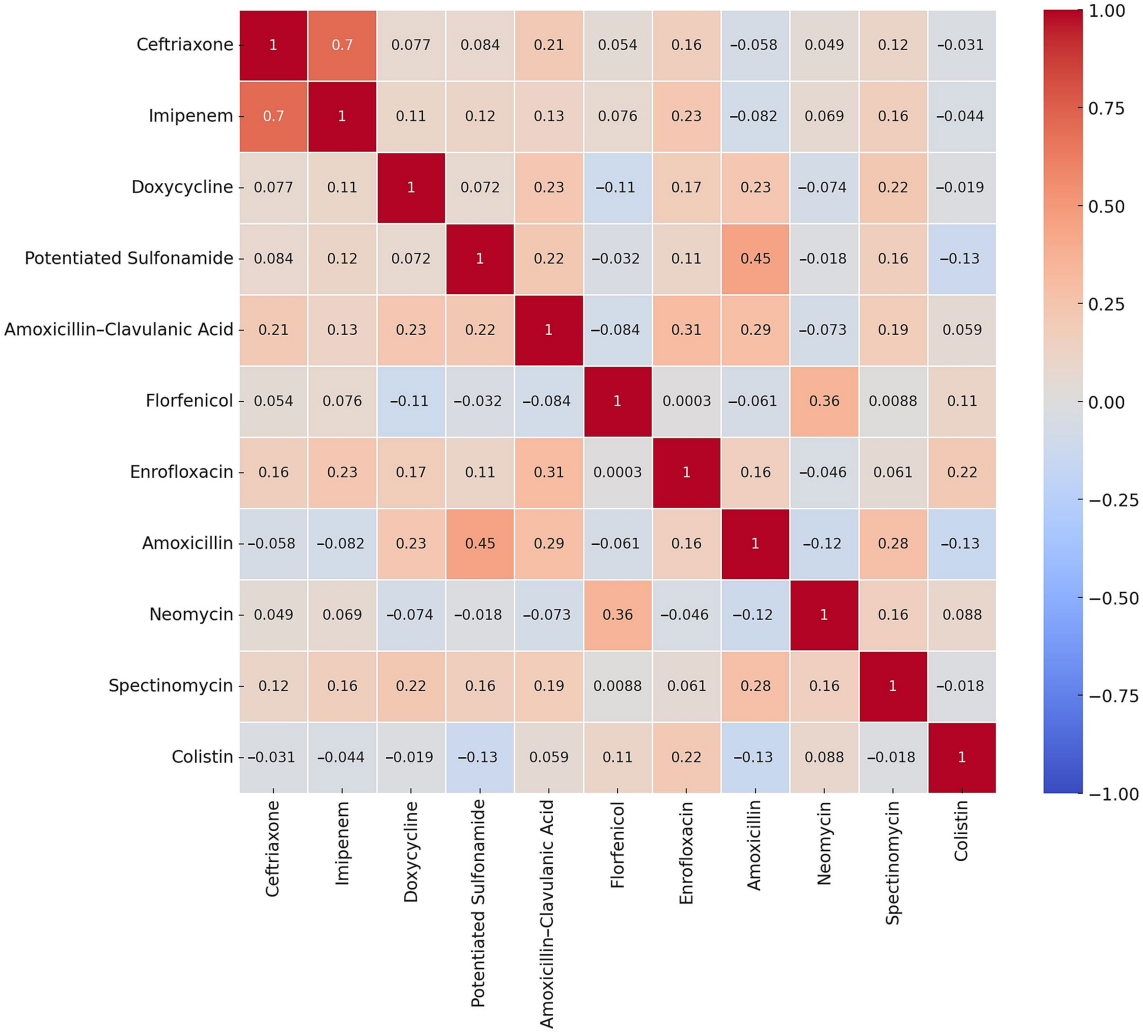
Heatmap depicting the resistance profile of *Escherichia coli* isolates (*n* = 134) from pigeons.

Pearson correlation analysis revealed varying degrees of association between antibiotic resistances. Using heatmap visualization (Figure 2), we identified stronger correlations that may indicate phenotypic co-occurrence of resistance traits. While these correlations do not imply causation or underlying genetic mechanisms, they may reflect patterns of co-selection, co-resistance, or cross-resistance—for instance, due to co-location of resistance genes on the same mobile genetic element or concurrent antimicrobial use.

The strongest positive correlation was observed between ceftriaxone and imipenem (*r* = 0.7). Other moderate positive correlations were noted between potentiated sulfonamide and amoxicillin (0.45). The most negative correlations were observed between amoxicillin and colistin (*r* = −0.13) and between potentiated sulphonamide and colistin (*r* = −0.13).

**Figure 3:**
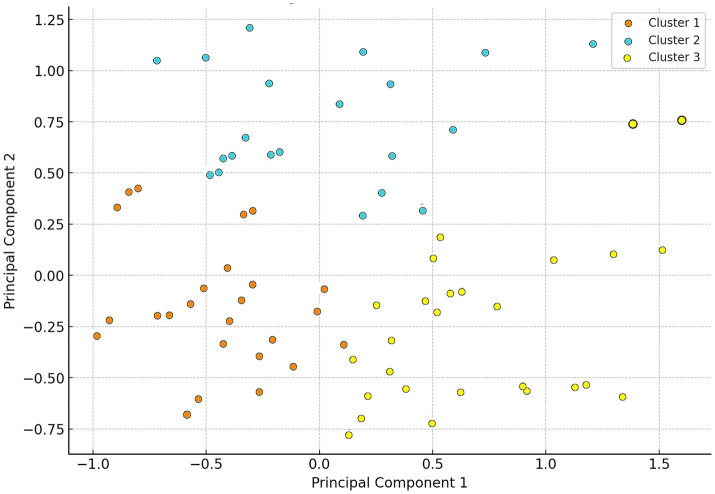
Cluster analysis of *Escherichia coli* isolates (*n* = 134) from pigeons based on resistance profiles. Cluster 1 members are marked in orange, Cluster 2 members in blue, and Cluster 3 members in yellow.

Cluster analysis identified three main groups (Figure 3), within which different dominant resistant agents were observed. In Cluster 1, neomycin was the most commonly resistant agent (95.2%), while doxycycline dominated in Cluster 2 (75.8%). In Cluster 3, neomycin was again the dominant resistant agent (92.3%).

### 3.3 Network analysis of co-resistance

**Figure 4:**
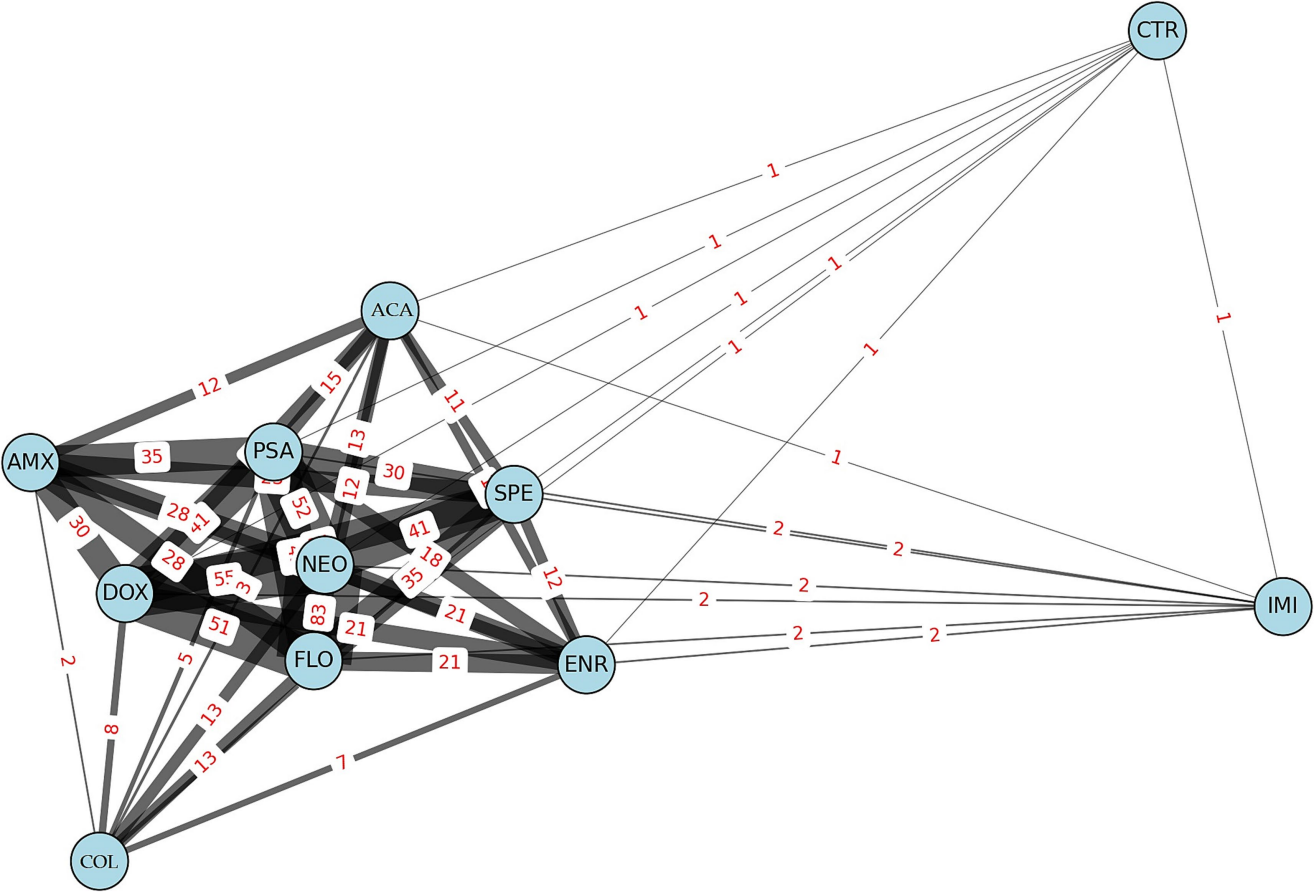
Network graph illustrates the frequency of connections between resistant *Escherichia coli* isolates (*n* = 134) from pigeons. AMX, amoxicillin; ACA, amoxicillin-clavulanic acid; CTR, ceftriaxone; COL, colistin; DOX, doxycycline; ENR, enrofloxacin; FLO, florfenicol; IMI, imipenem; NEO, neomycin; PSA, potentiated sulfonamide; SPE, spectinomycin.

Network graph analysis revealed the relationships and frequencies of connections between antibiotic resistances (Figure 4). In the network, line thickness indicates the frequency of co-resistance. Antibiotics are represented by light blue circles, the sizes of which are proportional to the number of resistant isolates associated with each agent. The most common associations were observed between neomycin, doxycycline, and florfenicol, suggesting potential cross-resistance between these antibiotics.

### 3.4 Predictive modeling and simulation

**Figure 5:**
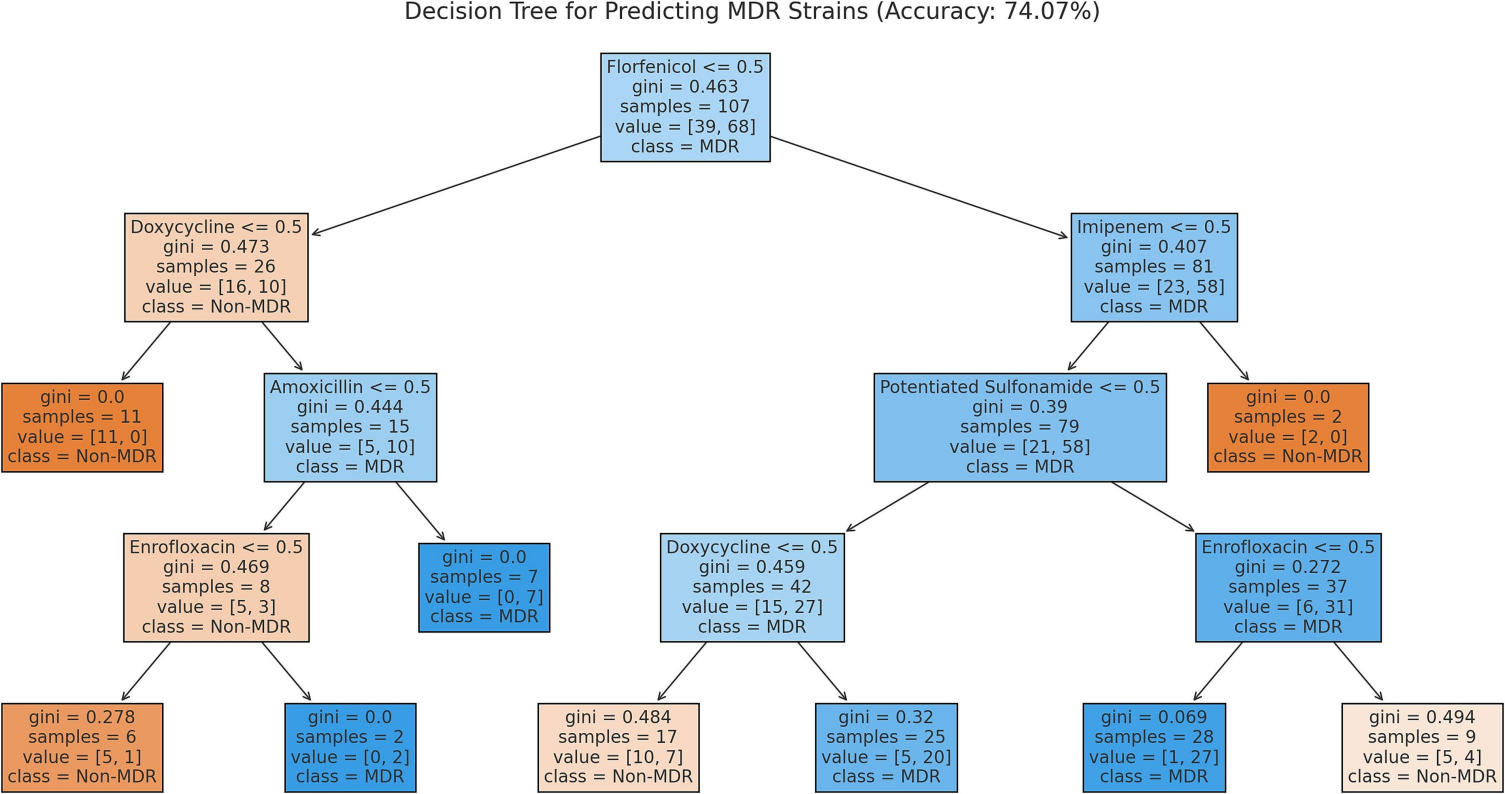
Decision tree model predicting the occurrence of multidrug-resistant strains among *Escherichia coli* isolates (*n* = 134) from pigeons.

The decision tree model allowed for the prediction of multidrug-resistant (MDR) strains (Figure 5). The model demonstrated that resistance to certain antibiotics was a stronger predictor of MDR status. Notably, resistance to neomycin, doxycycline, and amoxicillin played significant roles in identifying MDR strains.

**Figure 6:**
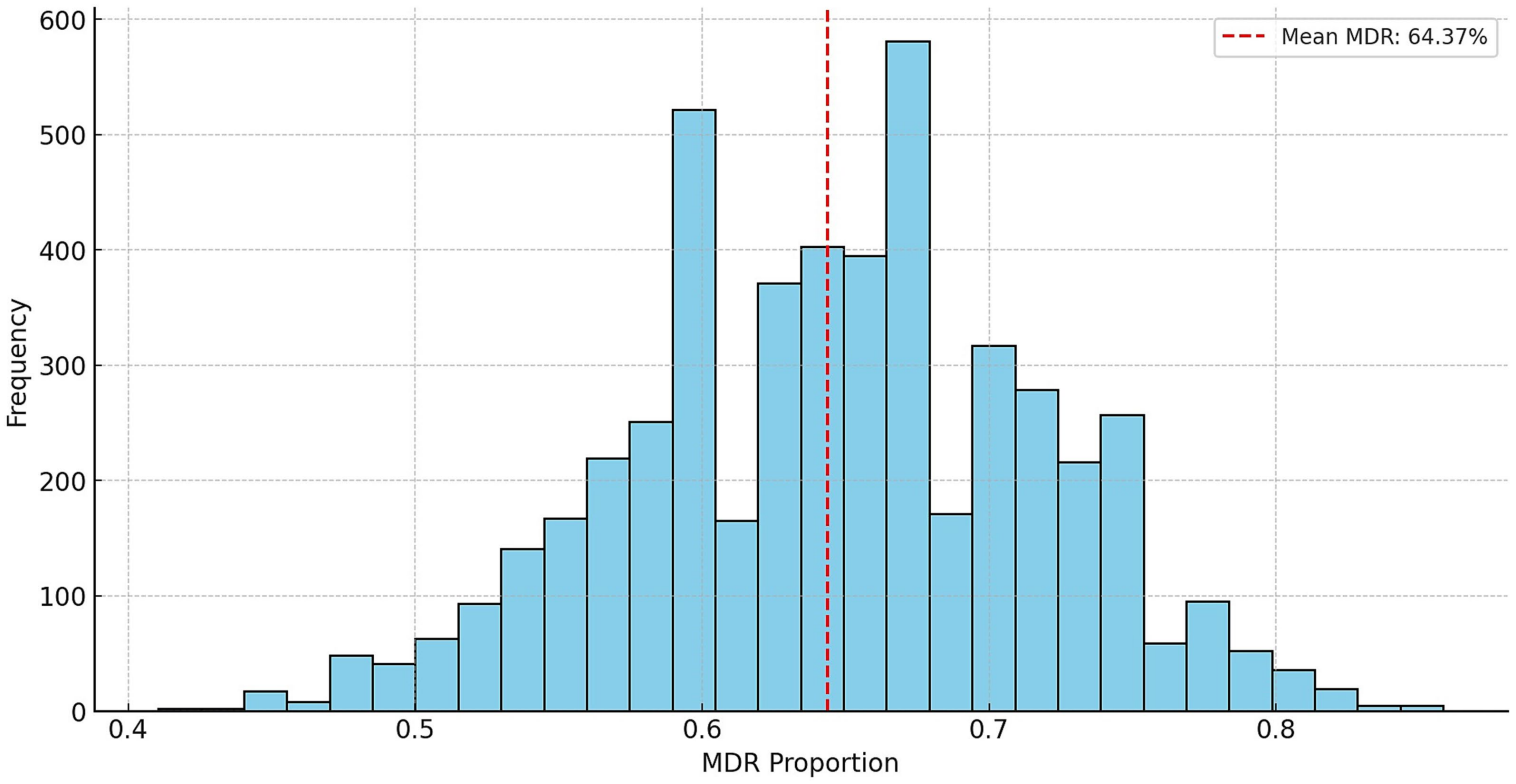
Stochastic prediction of multidrug-resistant occurrence in *Escherichia coli* isolates (*n* = 134) from pigeons using Monte Carlo simulation.

Stochastic modeling using Monte Carlo simulation was performed by randomly varying the original resistance ratios by ±20% (Figure 6). The average MDR occurrence rate was 64.4%, while the median value was 64.2%, indicating a symmetrical distribution. The standard deviation was 7.2%, indicating that the MDR rate generally ranged between 57.2 and 71.6%. The 95% confidence interval ranged from 50.0 to 77.6%, demonstrating the reliability of the simulation results.

### 3.5 Antimicrobial resistance prevalence and MIC values

Based on the determined minimum inhibitory concentration (MIC) values, a frequency table was created (Table 4) summarizing the results, including the clinical breakpoint for each agent. Additionally, the MIC_50_ and MIC_90_ values were calculated for the examined population. Among the agents tested, ceftriaxone and imipenem had MIC_90_ values below the clinical breakpoint, suggesting that at least 90% of infections could be treated effectively with these antibiotics. Considering the MIC_50_ values, amoxicillin, amoxicillin-clavulanic acid, ceftriaxone, colistin, enrofloxacin, and imipenem values were below the clinical breakpoint. Therefore, at least half of the samples retained susceptibility to these agents.

**Table 4:** Frequency distribution table of minimum inhibitory concentrations (MICs) for *Escherichia coli* isolates (*n* = 134) from pigeons, tested against antibiotics with established clinical breakpoints.

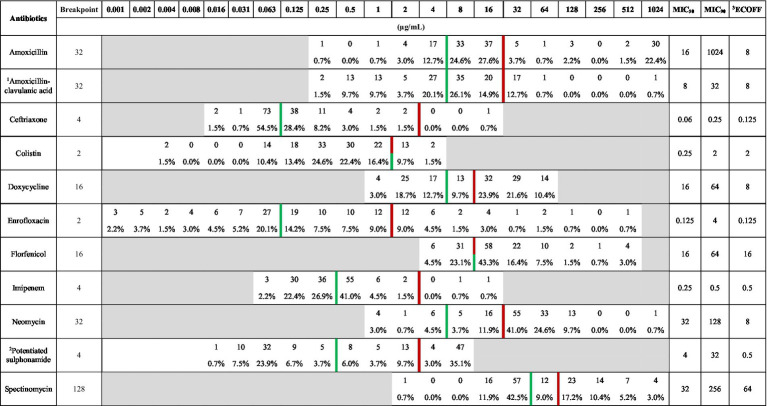

The upper row represents the frequency values, while the lower row indicates the corresponding percentage. Vertical red lines denote the clinical breakpoints, while vertical green lines represent the epidemiological cutoff values (ECOFF) defined by the European Committee on Antimicrobial Susceptibility Testing (EUCAST).

^1^ratio 2:1; ^2^trimethoprim and sulfamethoxazole in a 19:1 ratio, ^3^Epidemiological cutoff values (ECOFF) defined by the European Committee on Antimicrobial Susceptibility Testing (EUCAST).

When comparing our results with the epidemiological cut-off values (ECOFF) established by the European Committee on Antimicrobial Susceptibility Testing (EUCAST), we found that, in the cases of ceftriaxone, colistin, and imipenem, at least half of the examined samples were presumably composed of wild-type strains. This comparison was included to differentiate between acquired resistance (non-wild-type) and intrinsic susceptibility, regardless of current clinical breakpoints. By assessing both phenotypic resistance and ECOFF-based wild-type status, we aimed to gain deeper insight into emerging resistance trends, even in isolates that are not yet clinically categorized as resistant. Such information is critical for early detection of resistance development and for epidemiological surveillance within a One Health framework.

Detailed MIC values and isolate-specific data are available in Supplementary material.

**Figure 7:**
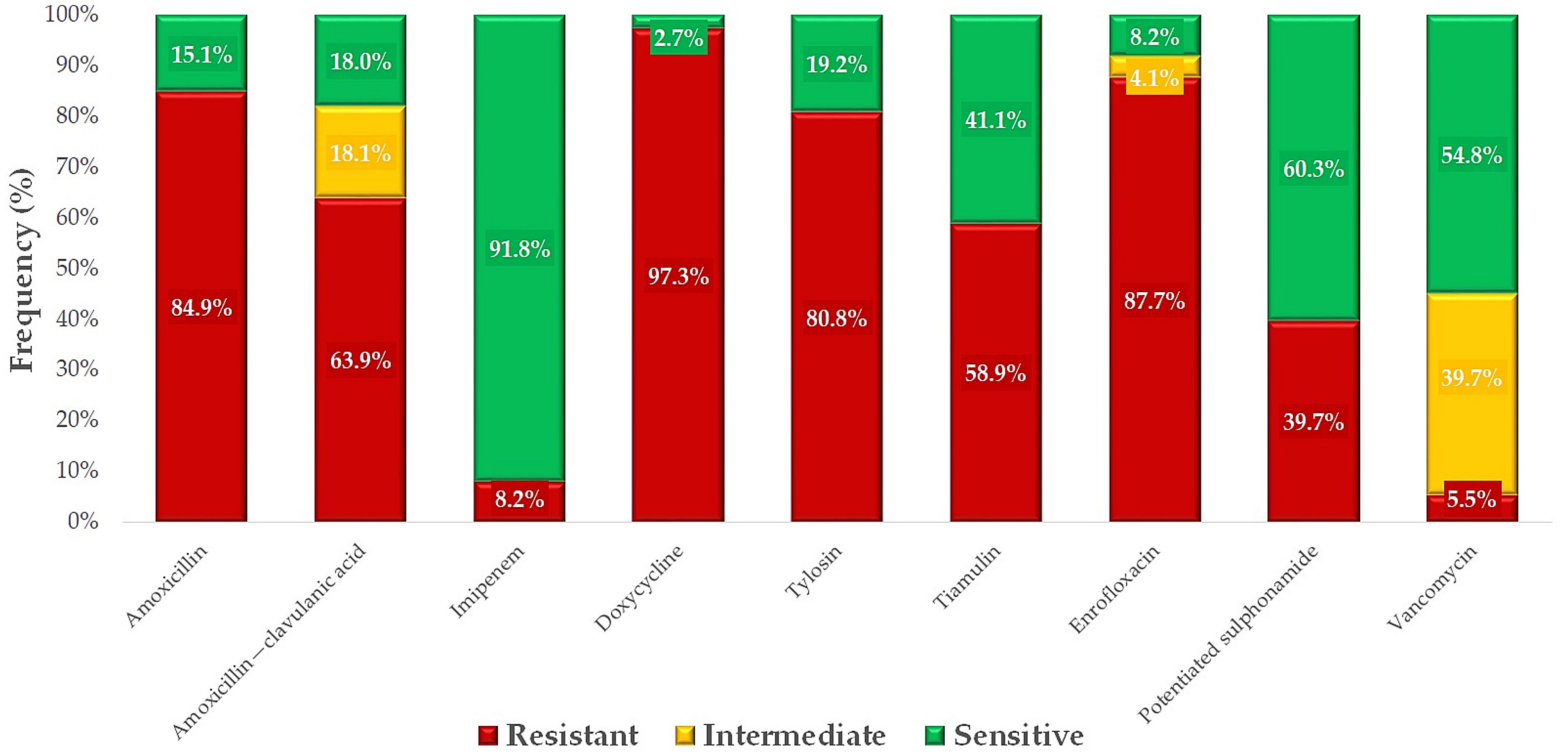
Antibiogram of *Escherichia coli* strains (*n* = 134) isolated from pigeons against antibiotics of public and animal health importance.

The ratio of resistant and susceptible strains was determined for each antimicrobial agent (Figure 7). The highest levels of resistance were observed against neomycin (76.1%), an aminoglycoside, and florfenicol (72.4%), a phenicol compound. In contrast, trimethoprim-sulfamethoxazole, a potentiated sulfonamide combination, retained its effectiveness, with no resistant strains identified. Minimal resistance was detected to ceftriaxone (0.7%), a third-generation cephalosporin, and imipenem (1.5%), a carbapenem.

### 3.6 Multidrug resistance patterns

The prevalence of MDR, XDR, and PDR strains were also assessed. MDR strains were predominant among the samples, with a proportion of 65.7% (88 strains). XDR strains accounted for only 4.5% (6 strains), while PDR strains were even rarer, with a prevalence of just 1.5% (2 strains).

### 3.7 Comparison with human resistance data

**Figure 8:**
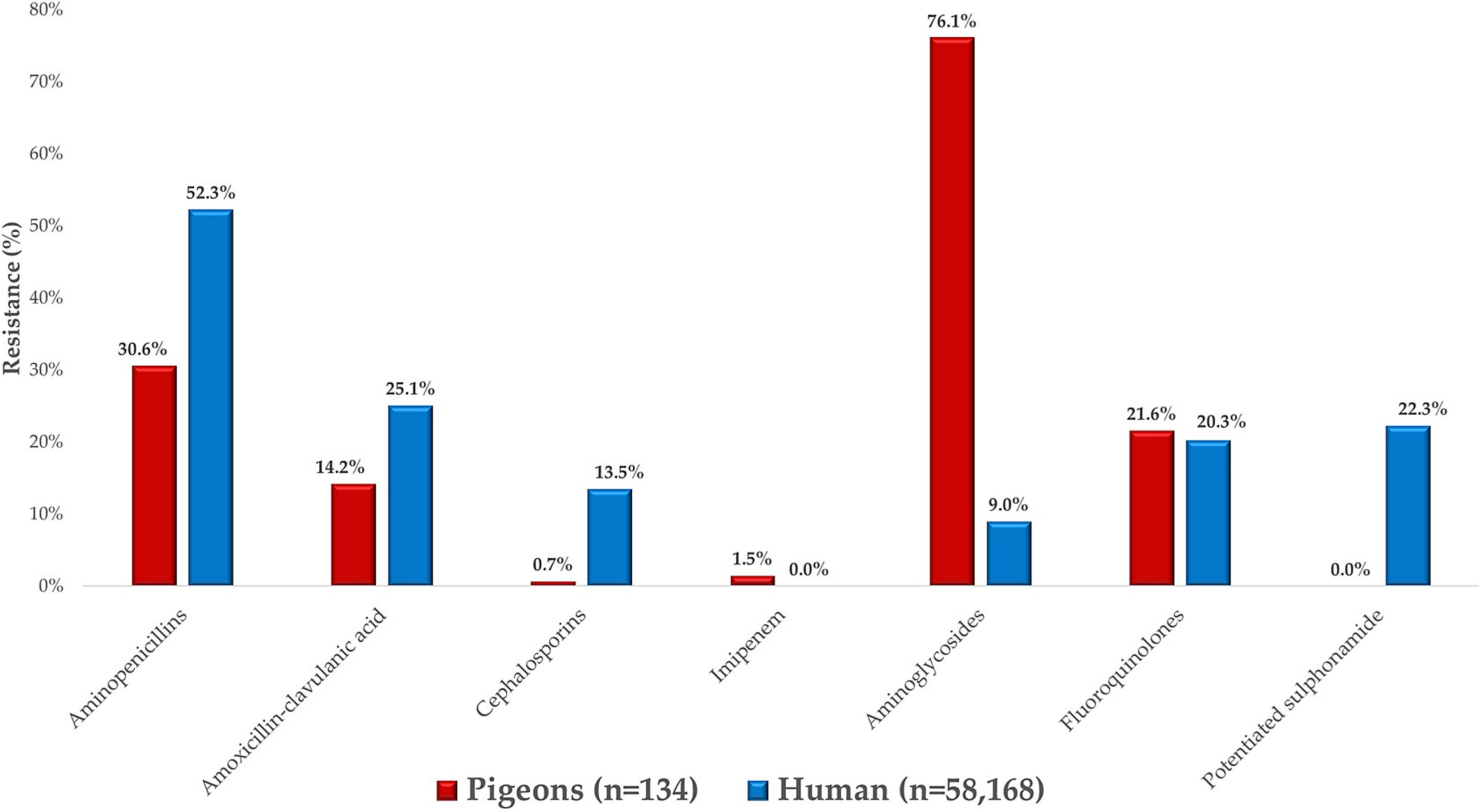
Comparison of *Escherichia coli* strains isolated from pigeons with human public health resistance data.

We compared resistance profiles of *E. coli* isolates from pigeons with human clinical resistance data obtained from the Hungarian National Public Health Service (Figure 8), in line with the study’s objective to explore potential overlaps in resistance patterns under the One Health framework. Regarding the beta-lactam class, significantly lower resistance levels were found in pigeon isolates compared to human clinical strains, which likely reflects differences in antibiotic exposure patterns. An exception was observed for imipenem, where a 1.5% resistance rate was detected in pigeon isolates, while no resistance was recorded in the corresponding human data. This finding may reflect sporadic environmental selection pressure or horizontal gene transfer events, although further molecular evidence would be needed to support this.

Fluoroquinolone resistance was similarly distributed between veterinary and human health samples, whereas no resistant strains were identified for potentiated sulfonamide. In contrast, the situation was much worse for aminoglycosides in veterinary samples, where the majority of strains were resistant.

## 4 Discussion

In the present study, we conducted a comprehensive analysis of the antimicrobial resistance of 134 *E. coli* strains isolated from pigeons using various statistical methods and stochastic modeling. Most of the samples originated from the Southern Great Plain region, where racing pigeons, as well as young adult and older breeding birds, were predominant.

The high prevalence of MDR strains in this population is particularly alarming, as 65.7% of the isolates (88 strains) met the MDR criteria. Additionally, the proportion of XDR strains was 4.5% (6 strains), while PDR strains were detected in 1.5% (2 strains) of the cases. These findings indicate that a large proportion of the examined isolates are resistant to multiple antibiotic classes, with a small but noteworthy fraction exhibiting extreme resistance phenotypes. In contrast, Karim et al. reported a notably lower MDR prevalence of 23.8% among *E. coli* isolates from pigeons in Bangladesh. In their study, most strains exhibited resistance to amoxicillin (61.9%), ampicillin (71.4%), erythromycin (61.9%), and tetracycline (52.4%) (62). The higher MDR rate observed in our study (65.7%) may reflect regional differences in antibiotic use practices, sampling methodologies, or the health status of the pigeon populations examined. These differences underscore the importance of localized AMR surveillance and the need to interpret such results within specific ecological and veterinary contexts. The authors attributed these patterns to the uncontrolled, empirical use of antibiotics, lack of professional veterinary oversight, and poor biosecurity among backyard and smallholder pigeon farms. Their study also noted statistical associations between resistance patterns, which may reflect underlying cross-resistance or co-selection processes. In our study, similar correlations were observed between certain antibiotics, particularly those with overlapping mechanisms of action or frequent co-application in veterinary practice. While such associations do not directly confirm biological mechanisms, they can provide a basis for hypothesizing potential resistance linkages, warranting further molecular investigation. Furthermore, they highlighted that antimicrobial resistance in pigeons may be amplified by environmental exposure to contaminated feed or water and close proximity to human settlements.

These findings support the hypothesis that the high occurrence of MDR among our pigeon isolates is likely linked to undocumented or uncontrolled antimicrobial use, combined with the absence of formal treatment records and limited veterinary intervention in the pigeon sector.

Although we were unable to obtain direct antimicrobial usage data, our results are consistent with resistance patterns in comparable husbandry systems.

Based on our results, the MIC_90_ values for ceftriaxone (0.25 μg/mL) and imipenem (0.5 μg/mL) were below the clinical breakpoint. This suggests that at least 90% of the population remains susceptible to these antibiotics. Regarding the MIC_50_ values, amoxicillin (16 μg/mL), amoxicillin-clavulanic acid (8 μg/mL), ceftriaxone (0.06 μg/mL), colistin (0.25 μg/mL), enrofloxacin (0.125 μg/mL), imipenem (0.25 μg/mL), and spectinomycin (32 μg/mL) demonstrated that at least half of the population retained sensitivity. In a Belgian study, MIC₅₀ and MIC₉₀ values for enrofloxacin were reported as <0.25 μg/mL and 32 μg/mL, respectively, in *E. coli* isolates from racing pigeons (63). The authors attributed the high MIC₉₀ value to the frequent prophylactic or empirical use of antibiotics, particularly fluoroquinolones like enrofloxacin, in racing pigeons, which may have contributed to the development and spread of resistant strains. In contrast, our study found lower MIC₅₀ and MIC₉₀ values (0.125 μg/mL and 8 μg/mL, respectively), possibly reflecting differences in antibiotic usage patterns. Notably, pigeon keepers in our study did not maintain records of antibiotic use and were reluctant to disclose such information, suggesting less widespread or undocumented antibiotic administration.

These MIC values are summarized in Table 1. When compared with the EUCAST ECOFFs, the MIC₅₀ values for ceftriaxone, colistin, and imipenem suggest that at least half of the isolates are likely wild-type, i.e., lacking acquired resistance mechanisms. This highlights the therapeutic potential of these agents and emphasizes the importance of preserving their efficacy through responsible use.

In our study, 65.7% of the *E. coli* strains were identified as multidrug-resistant (MDR), with resistance to florfenicol, amoxicillin, doxycycline, and imipenem contributing most significantly to the MDR phenotype. Notably, no resistance was detected against the tested potentiated sulphonamide (trimethoprim-sulfamethoxazole), suggesting its continued effectiveness in this context. A study by Carvalho et al. (64) reported similar patterns, where *E. coli* isolates from racing pigeons showed multidrug resistance to chloramphenicol, doxycycline, and trimethoprim-sulfamethoxazole, which supports the relevance of these compounds in resistance co-selection mechanisms.

Regarding fluoroquinolone resistance, a survey conducted in the Czech Republic reported 5.0% resistance to ciprofloxacin in *E. coli* isolated from urban pigeons (65), while a Belgian study documented a 13.0% resistance rate to enrofloxacin among isolates from racing pigeons (63). In contrast, our results demonstrated a 21.6% resistance rate to enrofloxacin, which may reflect differences in antimicrobial usage intensity, treatment regulation, and veterinary control across regions and pigeon-keeping systems.

A Spanish survey reported 48.7% resistance to amoxicillin-clavulanic acid, 97.6% to ceftazidime, and 51.4% to tetracycline, while all strains were sensitive to imipenem and potentiated sulfonamide (66). However, we found resistance rates of 14.2% for amoxicillin-clavulanic acid, 0.7% for ceftriaxone, 1.5% for imipenem, 56% for doxycycline, and no resistance to potentiated sulfonamide.

A Polish study reported resistance proportions of 63% for amoxicillin, 8% for amoxicillin-clavulanic acid, 22% for enrofloxacin, 74% for doxycycline, 2% for colistin, 53% for potentiated sulfonamide, 8% for florfenicol, and 15% for neomycin (67). In comparison, our results showed proportions of resistant isolates of 30.6, 14.2, 21.6, 64.0, 11.2, 0, 72.4, and 76.1%, respectively, for the same agents.

The correlation analysis demonstrated varying degrees of association between antibiotics. The strongest positive correlation was observed between ceftriaxone and imipenem (*r* = 0.7). While this correlation may reflect shared mechanisms of action, it is more plausibly explained by biological processes such as co-selection through mobile genetic elements. For example, resistance genes to multiple antibiotic classes can be co-located on plasmids, allowing for the simultaneous selection and maintenance of multidrug-resistant strains. Additionally, efflux pump systems such as AcrAB-TolC in *E. coli* are known to extrude a wide variety of structurally unrelated antibiotics, including β-lactams and fluoroquinolones, thereby contributing to correlated resistance patterns. Other potential contributors include porin mutations, which reduce membrane permeability and can lead to cross-class resistance. These findings underscore the need for further molecular investigations to confirm the underlying mechanisms. The positive correlation between potentiated sulfonamide and amoxicillin (*r* = 0.45) may indicate shared resistance dissemination pathways, such as co-selection or co-resistance via mobile genetic elements, rather than direct co-administration. These associations could arise from plasmids carrying multiple resistance genes or efflux systems that affect both classes. Conversely, negative correlations, such as those between potentiated sulfonamide and colistin (*r* = −0.13) or amoxicillin and colistin (*r* = −0.13), are due to the fact that these antibiotics work through different mechanisms or are applied under different conditions. Another study suggested that antibiotic resistance observed in *E. coli* strains isolated from pigeons is closely related to antibiotic use in humans, reinforcing the potential role of pigeons as reservoirs of resistance (68). These findings highlight the potential role of shared resistance mechanisms in shaping correlated resistance patterns among antibiotics. While the observed correlations do not establish causality, they suggest that co-selection via mobile genetic elements, efflux systems (e.g., AcrAB-TolC), and porin mutations may contribute to the concurrent resistance observed in our pigeon-derived *E. coli* isolates.

Although not directly proven in this study, the presence of multidrug-resistant *E. coli* strains in pigeons, particularly in urban settings where close contact with humans is common, raises concerns regarding their potential as zoonotic reservoirs. Further genomic studies would be needed to substantiate transmission dynamics and assess public health implications.

The cluster analysis identified three main groups, each containing different dominant resistant agents. The dominance of neomycin in both Cluster 1 and Cluster 3 (95.2 and 92.3%, respectively) and doxycycline in Cluster 2 (75.8%) suggests that these agents are particularly common among the examined pigeon populations. This overlap in neomycin resistance between clusters may reflect the ubiquitous nature of aminoglycoside resistance, which limits its discriminatory power in unsupervised clustering. Such overlap could indicate that the clustering algorithm—despite using a robust method (Jaccard distance and Ward’s linkage), did not fully separate resistance profiles where highly prevalent traits dominate. This limitation has been acknowledged, and future studies could explore alternative clustering strategies or the application of feature weighting to improve resolution.

Neomycin resistance is likely due to the widespread use of aminoglycoside antibiotics, while doxycycline resistance may be explained by the overexpression of efflux pumps or the presence of ribosomal protection proteins. A Chinese study demonstrated that multiple antibiotic resistance genes were present in *E. coli* strains isolated from pigeons, indicating a high prevalence of cross-resistance (69). In our study, the high resistance rate to doxycycline (56.0%) may be attributed to the widespread use of tetracyclines in veterinary medicine. Doxycycline belongs to this class, and resistance can emerge through common mechanisms such as efflux pumps, ribosomal protection proteins, or enzymatic inactivation. Moreover, tetracycline resistome is among the most diverse and widespread, with ARGs such as *tet(A)* and *tet(B)* commonly associated with mobile genetic elements. These factors could support cross-resistance between doxycycline and other tetracyclines, contributing to the high resistance observed in our isolates.

The network graph analysis provided further insight into the co-resistance patterns among the antibiotics. Notable associations were observed between neomycin, doxycycline, and florfenicol, indicating potential cross-resistance, particularly if these antibiotics are frequently used together or alternately. The decision tree model revealed that resistance to neomycin, doxycycline, and amoxicillin played significant roles in predicting MDR strains. This finding aligns with the results of the cluster analysis, where these antibiotics were identified as the most dominant resistant agents.

Resistance to several antibiotics showed overlapping trends between animal-derived and human *E. coli* isolates. For instance, both groups demonstrate high resistance to aminoglycosides and relatively low resistance to third-generation cephalosporins such as ceftriaxone. This pattern suggests that despite different sources, similar selective pressures—such as the use of related antibiotics in both veterinary and human medicine—may be contributing to comparable resistance phenotypes (70). The bootstrap analysis estimated the average MDR prevalence at 64.4%, with a 95% confidence interval ranging from 50.0 to 77.6% (Figure 6), reflecting substantial variability and uncertainty in the sampled pigeon population.

This high occurrence underscores the potential biological risk posed by MDR *E. coli* in avian populations, especially in the absence of documented antimicrobial use and surveillance. While similar patterns of increasing resistance have been reported in poultry farming systems (71), our results do not directly predict future trends, but rather emphasize the urgency for systematic monitoring and control under the One Health framework.

When comparing resistance profiles, we observed that veterinary isolates (from pigeons) demonstrated significantly lower resistance to β-lactam antibiotics, particularly ampicillin and ceftriaxone, compared to human isolates. However, similar resistance patterns were found for aminoglycosides, where resistance proportions were comparable across both datasets. This suggests that while overall resistance levels were lower in animal isolates, certain shared resistance trends may reflect overlapping antimicrobial use or environmental exposure. However, an exception was imipenem, where 1.5% of veterinary isolates were resistant, while no resistant strains were detected in public health samples. These findings are particularly notable given that resistance to potentiated sulfonamides is often more common in avian *E. coli* isolates, as reported in several international studies. The absence of resistance in our pigeon isolates may reflect limited or more prudent use of this antibiotic class in this species or region, although further investigation would be needed to confirm this. These findings highlight a growing divergence between veterinary and human antibiotic resistance profiles. These discrepancies underline the growing divergence between veterinary and human antibiotic resistance profiles, which may partly reflect the differential usage patterns of antimicrobial agents that are specific to either human or veterinary medicine. This underscores the urgent need for integrated One Health surveillance strategies that bridge gaps between veterinary and human public health sectors.

This study has several limitations that should be acknowledged. First, although the role of antimicrobial use in the development of MDR phenotypes is widely recognized, flock-level data on antibiotic usage was unavailable, due to the lack of documented treatment records and the reluctance of pigeon keepers to disclose such information. This limited our ability to draw direct associations between resistance patterns and antimicrobial exposure.

Second, the study design was cross-sectional, providing a snapshot of resistance at a single point in time. As such, temporal trends and causality cannot be inferred. Longitudinal studies are needed to monitor resistance dynamics over time and assess the impact of potential interventions.

Third, resistance mechanisms were inferred from phenotypic data and statistical correlations. Although suggestive patterns were observed, no molecular characterization (e.g., PCR, whole-genome sequencing) was performed to identify specific resistance genes or mobile genetic elements. Future studies incorporating genomic approaches would provide deeper insights into co-resistance, cross-resistance, and horizontal gene transfer.

Fourth, although isolates were collected from multiple geographic regions across Hungary, sampling was not strictly random, and the sample size was pragmatically determined based on field logistics rather than statistical power calculations. This may introduce selection bias and limit the generalizability of findings.

Finally, the comparative analysis with human resistance data, while valuable, is constrained by differences in surveillance methodologies and antibiotic panels used between human and veterinary sectors. Harmonized One Health surveillance frameworks would enhance such comparative efforts in the future.

In conclusion, our findings highlight that MDR *E. coli* strains are prevalent among pigeons, and simulations suggest that this prevalence may continue to increase. To avoid the further spread of cross-resistance, ongoing monitoring and more detailed investigations into the co-application of different antibiotics are necessary. Additionally, farmer education programs should be implemented to emphasize the importance of accurate and transparent treatment records, particularly in non-commercial pigeon keeping systems. These findings should be interpreted within a One Health framework, recognizing that resistance in avian species may impact broader ecosystems, animal welfare, and ultimately human health.

## 5 Conclusion

This study demonstrates that 65.7% of *E. coli* isolates from domestic pigeons in Hungary exhibit multidrug resistance (MDR), with additional identification of XDR (4.5%) and PDR (1.5%) profiles. These findings raise important concerns regarding antimicrobial use in pigeon populations and potential public health risks. Statistical analyses revealed consistent co-occurrence patterns among resistance traits, particularly involving neomycin, doxycycline, and florfenicol. While these associations suggest the potential for cross-resistance or co-selection, no molecular mechanisms were investigated, and the findings remain observational. Bootstrap-based prevalence estimates highlighted considerable uncertainty, indicating the need for enhanced sampling and surveillance strategies. Although no direct prediction of future trends was made, the observed MDR burden underscores the importance of implementing antimicrobial stewardship in pigeon husbandry. Under the One Health framework, routine resistance monitoring, cross-sectoral data integration, and molecular-level investigations are needed to clarify resistance drivers and inform control strategies.

## Data Availability

The original contributions presented in the study are included in the article/Supplementary material, further inquiries can be directed to the corresponding author.
